# New algorithm improves fine structure of the barley consensus SNP map

**DOI:** 10.1186/1471-2164-12-407

**Published:** 2011-08-10

**Authors:** Jeffrey B Endelman

**Affiliations:** 1 Department of Crop and Soil Sciences, Washington State University, 16650 State Route 536, Mount Vernon, WA 98273, USA

## Abstract

**Background:**

The need to integrate information from multiple linkage maps is a long-standing problem in genetics. One way to visualize the complex ordinal relationships is with a directed graph, where each vertex in the graph is a bin of markers. When there are no ordering conflicts between the linkage maps, the result is a directed acyclic graph, or DAG, which can then be linearized to produce a consensus map.

**Results:**

New algorithms for the simplification and linearization of consensus graphs have been implemented as a package for the R computing environment called DAGGER. The simplified consensus graphs produced by DAGGER exactly capture the ordinal relationships present in a series of linkage maps. Using either linear or quadratic programming, DAGGER generates a consensus map with minimum error relative to the linkage maps while remaining ordinally consistent with them. Both linearization methods produce consensus maps that are compressed relative to the mean of the linkage maps. After rescaling, however, the consensus maps had higher accuracy (and higher marker density) than the individual linkage maps in genetic simulations. When applied to four barley linkage maps genotyped at nearly 3000 SNP markers, DAGGER produced a consensus map with improved fine structure compared to the existing barley consensus SNP map. The root-mean-squared error between the linkage maps and the DAGGER map was 0.82 cM per marker interval compared to 2.28 cM for the existing consensus map. Examination of the barley hardness locus at the 5HS telomere, for which there is a physical map, confirmed that the DAGGER output was more accurate for fine structure analysis.

**Conclusions:**

The R package DAGGER is an effective, freely available resource for integrating the information from a set of consistent linkage maps.

## Background

The need to integrate information from multiple linkage maps into a consensus map is a long-standing problem in genetics [1,2]. Consensus maps have been developed for many crops, including wheat [3], sorghum [4], and potato [5]. Within the barley research community alone, at least seven consensus maps have been published in the past six years [6-12].

Wenzl et al. [7] differentiated between two broad strategies for constructing consensus maps. In the traditional approach, the consensus map is determined directly from the genotypic data, using extensions of the maximum-likelihood methods developed for single populations [13]. While this approach was effective for many years, it has not always produced consistent and timely results as marker densities have continued to increase [7,14]. These limitations have spurred innovation in a second ("synthetic" [7]) strategy, in which the consensus map is generated from the linkage maps without recourse to the original genotypic data.

**Figure 1:**
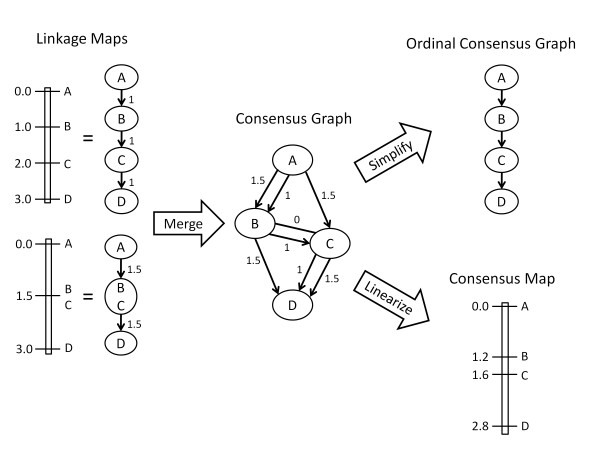
**Diagram of the DAGGER package**. Linkage maps are first converted into unbranched directed graphs, where each vertex is a marker bin and each edge length is the distance of the corresponding map interval. After merging the linkage maps, the consensus graph is checked for cycles, which represent ordering conflicts. Provided there are none, the consensus graph can be simplified to generate an ordinal consensus graph, which contains only those edges needed for ordinal equivalency with the linkage maps. The consensus graph can also be linearized to produce a consensus map, using either linear or quadratic programming (latter is shown).

It was Yap et al. [15] who first recognized that the complex ordinal relationships present in a series of linkage maps can be exactly represented as a directed graph (Figure 1). Each graph represents a single chromosome and each vertex in the graph represents a bin of markers. Provided there are no ordering conflicts between the linkage maps, the ordinal relationships in the directed graph are equivalent to those in the linkage maps. In other words, there is a path from vertex *v *to vertex *w *in the graph if and only if bin *v *comes before bin *w *in one of the linkage maps.

Wu et al. [14] have capitalized on this graph-theoretic formulation in their software MergeMap [16], a free resource for constructing consensus maps. MergeMap contains an efficient algorithm for resolving ordering conflicts between linkage maps, which appear as cycles in the consensus graph. The removal of these cycles produces a directed acyclic graph, or DAG, which MergeMap then simplifies and linearizes to produce a consensus map.

Close et al. [11] used MergeMap to integrate four barley linkage maps genotyped at nearly 3000 SNP markers. The resulting consensus SNP map has been used for association mapping [17-19] by members of the Barley Coordinated Agriculture Project (Barley CAP) [20]. In addition to the consensus map, Close et al. [11] published consensus graphs for each of the seven barley chromosomes. While examining the fine structure of the hardness locus on the 5HS telomere, which has been sequenced [21], several unphysical orderings were detected in the consensus graph that are not present in any of the linkage maps.

The discovery that the simplified consensus graphs produced by MergeMap are not ordinally equivalent to the linkage maps prompted the development of new algorithms for the simplification and linearization of consensus graphs. These algorithms have been implemented as a package for the R computing environment [22] called DAGGER [23]. To validate DAGGER, a new barley consensus SNP map was constructed and compared with the results of Close et al. [11]. The performance of DAGGER was also evaluated with simulated data.

## Results and Discussion

### Constructing the consensus graph

Given a list of linkage maps, DAGGER builds up the consensus graph sequentially. The first two linkage maps are merged to create a consensus graph, which is then merged with the next linkage map, and so on. The merging algorithm proceeds from the first to the last bin in the linkage map *M *that is to be integrated with graph *G*. Let *S_v _*be the set of vertices in *G *that contain one or more markers in bin *v *of *M*. For each vertex *w *in *S_v_*, if all of the markers in *w *are contained in *v*, then *w *remains intact. If only some of the markers in *w *are in *v*, then *w *is split into two vertices: *w*_1 _contains the common markers between *v *and *w*, and *w*_2 _contains the remaining markers in *w*. All of the edges directed in and out of *w *are replicated for *w*_1 _and *w*_2_. Vertex *w*_1 _also receives new edges directed in and out of it according to the immediately proximal and distal bins in *M*. Any markers in *v *that were not present in *G *are added as a new vertex with appropriate edges. When completed, the consensus graph contains a directed edge for every map interval between adjacent bins in the linkage maps (Figure 1).

During construction of the consensus graph, DAGGER also keeps track of which markers were binned in the linkage maps. The map distance between binned markers is zero, and this information is needed to minimize the error between the consensus map and linkage maps (see below). Although depicted in the consensus graph (Figure 1), the zero-length edges are not part of its topology (i.e., they do not imply ordinal relationships).

DAGGER checks for ordering conflicts between the linkage maps by identifying the strongly connected components of the consensus graph. Two vertices *v *and *w *are strongly connected if there is a path from *v *to *w *and from *w *to *v*. In the context of markers, that would mean the markers in *v *mapped before those in *w*, which in turn mapped before those in *v*; this represents an inconsistency between the linkage maps.

Identifying the strongly connected components of a directed graph is a standard exercise in computer science [24]. DAGGER performs two depth-first searches, one on the reverse graph *G^R ^*(formed by reversing the edges in *G*), and then on *G *itself. The traversal of *G^R ^*generates a topologically sorted list of the vertices. By conducting the depth-first search of *G *in this order, the algorithm identifies the strongly connected components of *G*.

DAGGER will only proceed with graph simplification and linearization if the marker order between linkage maps is consistent. To facilitate manual curation of errors in the linkage maps, DAGGER will output the strongly connected components of *G *for visualization with the Graphviz dot software [25]. When the inconsistency is due to a small misplacement, this visual will indicate where to look in the genotypic data to resolve the ordering conflict. For more complex and long-distance inconsistencies, the conflict-resolution algorithm in MergeMap [14] is a valuable resource.

### Simplifying the consensus graph

In the absence of ordering conflicts, DAGGER will output the consensus graph for visualization in one of two forms. The first option is to display the graph with all of the distance information used to generate the consensus map. The second option is to simplify the graph so that it conveys the ordering of the markers but not their distances, which potentially allows many edges to be removed (Figure 1).

The ordinal consensus graph produced by DAGGER contains only those edges that are needed to satisfy the equivalency property stated in the introduction, namely, that there exists a path from vertex *v *to vertex *w *if and only if bin *v *mapped before bin *w *in one of the linkage maps. The edge reduction proceeds by first using the topologically sorted vertex list from the depth-first search of *G^R ^*to determine, for each vertex *v*, the list of all vertices reachable from *v*. Next, the edges for each vertex are considered for elimination according to the topological sort order of the vertices to which they are directed. Let *Q *be a topologically sorted list of the vertices to which there are directed edges from vertex *v*. If vertex *Q_k _*is reachable from vertex *Q_j _*with *j < k*, then the edge to *Q_k _*is removed.

Following the edge reduction, DAGGER performs one other simplification without loss of ordinal information. Some of the marker bins that were split during the construction of the consensus graph, due to different distance estimates, may have the same pattern of inward and outward edges after the edge reduction. These vertices are combined and their markers put back in one bin.

### Linearizing the consensus graph

Whereas the ordinal consensus graph produced by DAGGER will not be invalidated by further research (provided the component linkage maps are correct), this is not true of the marker order in the consensus map. Because recombination distances are population-dependent, the relative order of markers that have not been mapped together in a single population cannot always be determined unambiguously.

DAGGER generates a consensus map by minimizing the "error" between the consensus map and the linkage maps. In mathematical terms, this error may be written as(1)

where *x*(*m*_1_) and *x*(*m*_2_) are the positions of markers *m*_1 _and *m*_2 _in the consensus map **x**, and *d_k_*(*m*_1_,*m*_2_) ≥ 0 is a distance interval from the *k*^th ^linkage map. When the exponent *α *= 1, DAGGER minimizes the *L*_1 _norm; when *α *= 2, the *L*_2 _norm is minimized. The outer sum in Equation 1 is over the linkage maps, and the inner sum is over all pairs of markers (*m*_1_,*m*_2_) in either the same or adjacent bins in the *k*^th ^linkage map.

Because this double summation exactly corresponds to the edges (both directed and zero-length) in the consensus graph, Equation 1 can be rewritten as a sum over edges with an appropriate weighting factor:(2)

Analogous to Equation 1, *x*(*v*) and *x*(*w*) are the positions of marker bins *v *and *w *in the consensus map (every vertex in the consensus graph becomes a bin in the consensus map), and *d*(*v, w*) ≥ 0 is an edge length. For Equation 1 to equal Equation 2, the weighting factor *q_v,w _*= (# markers in *v*) × (# markers in *w*).

In addition to having minimum error, it is desirable that the consensus map be entirely consistent with the consensus graph (and by extension the linkage maps) in terms of marker order. This means that for every directed edge *t *→ *u *in the consensus graph, the inequality *x*(*t*) ≤ *x*(*u*) is required for the consensus map. When this set of linear inequalities is combined with minimizing Equation 2, the constrained optimization problem solved by DAGGER can be written as(3)

It is straightforward to show that Equation 3 can be solved by linear (*α *= 1) or quadratic (*α *= 2) programming methods. Let **A **be the *p × n *adjacency matrix for the directed edges in the consensus graph (*p *edges, *n *vertices). This means that in correspondence with the *k*^th ^edge *t → u*, the *k*^th ^row of **A **contains -1 at the position for bin *t *and 1 at the position for bin *u*. The *r × n *adjacency matrix **B **for the zero-length edges is defined similarly, and the combined (*p*+*r*)*× n *adjacency matrix **C **is formed by stacking **A **on **B**. If the vector **d **contains the edge lengths *d*(*v, w*) corresponding to the rows of **A **(*d *> 0) and **B **(*d *= 0), then the error vector is **Cx **- **d **and the consistency constraints are **Ax **≥ **0**. For *α *= 2, Equation 3 is equivalent to the following quadratic program (QP):(4)

where the matrix **Q **= diag(**q**), and **q **is the vector of weights *q_v, w _*in Equation 3. By introducing *p*+*r *additional variables, denoted by **z**, Equation 3 with *α *= 1 is equivalent to the following linear program (LP) [26]:(5)

### Validation with simulated data

To test the performance of DAGGER, it was applied to simulated linkage maps generated from 1000 evenly spaced markers with a total map length of 100 cM. The results in Table 1 are for random parents with an average sequence identity of 70%, for which the expected number of markers per linkage map is 0.3 × 1000 = 300. Using more divergent parents led to maps with more markers, but the trends were the same.

**Table 1:** DAGGER performance on simulated data

	# Linkage maps					
	1	2	4	6	8	Method
# Markers	300 (1)	514 (2)	761 (1)	884 (1)	944 (1)	LP/QP

Map length, cM	99 (1)	82.9 (0.6)	70.9 (0.5)	61.6 (0.4)	56.4 (0.3)	LP
		87.7 (0.6)	73.9 (0.4)	66.2 (0.3)	61.6 (0.3)	QP

Mean absolute error, cM	4.0 (0.2)	3.4 (0.2)	2.6 (0.1)	2.4 (0.1)	2.3 (0.1)	LP
		3.1 (0.2)	2.4 (0.1)	2.2 (0.1)	2.0 (0.1)	QP

RMS error, cM	4.7 (0.2)	4.0 (0.2)	3.1 (0.1)	2.9 (0.1)	2.7 (0.1)	LP
		3.6 (0.2)	2.8 (0.1)	2.6 (0.1)	2.4 (0.1)	QP

Time, s	--	2.0 (0.0)	11.0 (0.1)	29.6 (0.2)	57.4 (0.4)	LP
		2.0 (0.0)	11.7 (0.1)	29.4 (0.2)	50.7 (0.4)	QP

The mean (and SE) are reported for 100 simulations of a 100 cM interval containing 1000 markers. Linkage maps were generated from doubled haploid populations of 200 offspring, using parents with 70% sequence identity. Results are shown for both the LP and QP methods of linearizing the consensus graph (error was calculated after rescaling).

The simulated data illustrate the primary benefit of integrating multiple linkage maps, which is that higher marker densities are possible. Table 1 shows that the average number of markers in each linkage map was indistinguishable from the expected value of 300. Consensus maps based on two linkage maps contained an average of 514 markers, and with eight linkage maps an average marker density of 944 out of 1000 was achieved.

An unusual feature of both the LP and QP linearization methods is their tendency to compress map intervals, whereas most consensus maps are inflated relative to the original linkage maps (e.g., [7,11]). As shown in Table 1, the amount of compression increased as more linkage maps were integrated. For two linkage maps, the LP and QP consensus maps were compressed by 17% and 12% respectively, while for eight linkage maps the compression was 44% (LP) and 38% (QP). The QP consensus maps had consistently less compression than the LP maps. For the simple example shown in Figure 1, where both linkage maps have a total length of 3.0 cM, one can verify analytically that the QP consensus map is compressed to 2.8 cM (the LP map is 2.5 cM). By default DAGGER rescales the consensus map so that its total length equals the mean length of the component linkage maps, but the user can also request the compressed map.

Table 1 shows that, in addition to increasing marker density, integrating more linkage maps tends to reduce the error between the consensus map and the simulated physical map. This was true regardless of whether error was measured with the *L*_1 _norm (mean absolute error) or *L*_2 _norm (root-mean-squared [RMS] error). With only one linkage map the average RMS error was 4.7 (SE 0.2) cM, while with eight linkage maps the average RMS error was 2.4 (SE 0.1) cM for the QP method. Using either norm, the QP maps had consistently less error than the LP maps (contrast: RMSE_QP _- RMSE_LP _= -0.3 cM < 0, p < 10^-4^).

### Validation with barley data

The original impetus for developing DAGGER came from analyzing the results of Close et al. [11], who used MergeMap [14] to integrate the information from four barley linkage maps genotyped at nearly 3000 SNP markers. These same linkage maps were used as input for DAGGER, and no ordering conflicts were detected. The ordinal consensus graphs for the seven barley chromosomes (Figures S1-S7, Additional Files 1, 2, 3, 4, 5, 6, and 7) and the QP consensus map (Table S1, Additional File 8) produced by DAGGER are available online.

**Figure 2:**
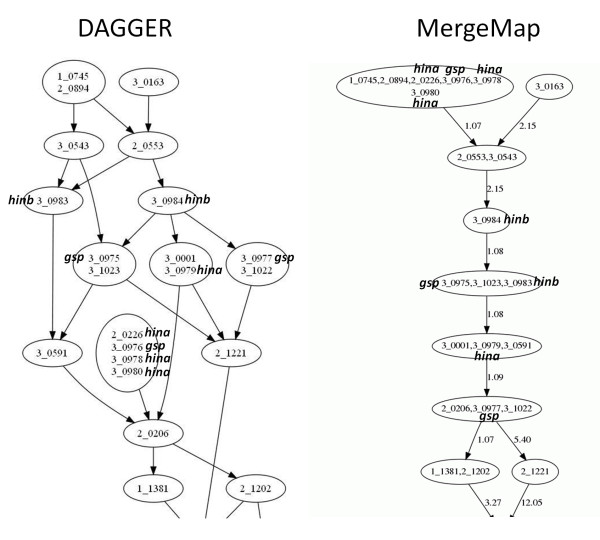
**Simplified consensus graphs of the barley 5HS telomere**. Marker labels are shown within each vertex, and markers for genes at the hardness locus are indicated (*hinb/hina/gsp*). On the left side of the figure is the ordinal consensus graph produced by DAGGER, in which there is a path from vertex *v *to vertex *w *if and only if *v *mapped before *w *in one of the linkage maps. On the right side is the simplified consensus graph produced by MergeMap. Comparison with Table 2, which contains the linkage maps for the 5HS telomere, confirms that the output from DAGGER is ordinally equivalent to the linkage maps, while the output from MergeMap is not. In particular, the simplified graph from MergeMap contains several unphysical relationships for the hardness locus markers.

Figure 2 compares the ordinal consensus graph from DAGGER and the simplified consensus graph from MergeMap at the 5HS telomere. The four linkage maps for this region are reproduced in Table 2. Nine of the markers are for genes at the barley hardness locus, for which there is a physical map [21]. The physical order of these genes is *hinb/hina/gsp *from the distal end.

**Table 2:** Barley linkage maps in the 5HS telomere region

Notes	Marker	SM	OWB	MB	HO
	3_0163		0		
	1_0745	0		0	
	2_0894	0		0	
	2_0553		2.15	1.07	
	3_0543	0		1.07	
*hinb*	3_0983			2.15	
*hinb*	3_0984	0	4.30		
*hina*	2_0226	0			
*hina*	3_0978	0			
*hina*	3_0979	0	5.38		
*hina*	3_0980	0			
*gsp*	3_0975	0	5.38	2.15	
*gsp*	3_0976	0			
*gsp*	3_0977		5.38		
	3_0001	0	5.38		
	3_1022		5.38		
	3_1023	0	5.38	2.15	
	3_0591	0		3.23	
	2_0206	1.09		3.23	
	1_1381	2.17			0
	2_1202	2.17		4.30	
	2_1221		10.78		

Abbreviations are SM = Steptoe × Morex, OWB = Oregon Wolfe Barley, MB = Morex × Barke, HO = Haruno Nijo × OUH602. Blank entries indicate the marker was not mapped in that population.

None of the ordinal information in the DAGGER graph violates this physical order. The only relationships among the *hinb/hina/gsp *markers in the DAGGER graph are that marker 3_0984(*hinb*) is distal to markers 3_0975(*gsp*), 3_0979(*hina*), and 3_0977(*gsp*). Table 2 shows that this information comes from the Oregon Wolfe Barley (OWB) linkage map, and that no other ordinal information for the hardness locus markers is present in the linkage maps.

MergeMap also captures this ordering, but in addition there are numerous relationships that are not present in the linkage maps. This is apparent from the unphysical ordering of the *hinb/hina/gsp *markers. MergeMap shows the markers 3_0976(*gsp*), 3_0978(*hina*), 3_0980(*hina*), and 2_0226(*hina*) as mapping distal to both *hinb *markers, and the marker 3_0975(*gsp*) is shown distal to 3_0979(*hina*). The order of these markers is indeterminate from the linkage maps, which is how they are portrayed by DAGGER. The unphysical relationships in the simplified graph from MergeMap arise because of the way markers are binned. MergeMap will potentially bin markers if they are binned in at least one of the linkage maps and if their order is indeterminate [14].

To appreciate the consequences of this rule, consider the vertex with six markers at the top of the MergeMap graph. Four of these markers are from the hardness locus and were only present in the Steptoe-Morex (SM) map: 3_0976(*gsp*), 3_0978(*hina*), 3_0980(*hina*), and 2_0226(*hina*). The other two markers in the vertex--1_0745 and 2_0894--are binned with the hardness locus markers in the SM linkage map (see Table 2). The markers 1_0745 and 2_0894 were also co-segregating in the Morex-Barke (MB) population, where they mapped distal to the *hinb *marker 3_0983. Because the aforementioned *hina *and *gsp *markers were binned with 1_0745 and 2_0894 in the SM linkage map, and because their relationship to 3_0983(*hinb*) is indeterminate from the linkage maps, MergeMap binned all six markers together. This creates the implication that the *hina *and *gsp *markers are distal to 3_0983(*hinb*), which is consistent with the linkage maps but not implied by them (and known to be false from the physical map [21]). As this example illustrates, the simplified graphs from MergeMap are not ordinally equivalent to the linkage maps and will potentially contain unphysical relationships even if the linkage maps do not. The simplified graphs from DAGGER are ordinally equivalent to the linkage maps and will not contain unphysical relationships provided the linkage maps are physically correct.

Table 3 shows the number of markers, number of marker bins, and amount of compression in the DAGGER consensus map (using the QP method). Because DAGGER linearizes the unsimplified consensus graph, whereas MergeMap linearizes a simplified graph, the consensus map from DAGGER had twice the number of marker bins (1886 vs. 942). Over the entire genome the DAGGER map was compressed by 13%, while the consensus map from MergeMap was inflated by 46%. The average correlation coefficient between the two maps was 0.95 on a scale of 10 cM, indicating a high degree of similarity.

**Table 3:** Barley consensus map statistics

		Mean Linkage Map	Consensus Map Length (% of Mean)		# Bins	
Chromosome	# Markers	Length (cM)	DAGGER	MergeMap	DAGGER	MergeMap
1H	341	141	88%	144%	242	117
2H	485	161	86%	148%	310	153
3H	475	174	85%	140%	320	149
4H	335	125	92%	143%	207	111
5H	535	198	87%	152%	315	174
6H	352	133	91%	154%	229	109
7H	417	167	86%	138%	263	129
Total	2940	1099	87%	146%	1886	942

The DAGGER map was generated with the QP method.

**Figure 3:**
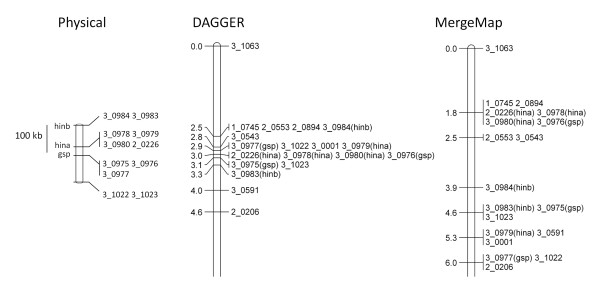
**Consensus maps of the barley 5HS telomere**. The rescaled consensus maps from DAGGER and MergeMap (distances in cM) are shown alongside a physical map of the hardness locus. The scale of the physical map relative to the consensus maps is arbitrary; the scale bar refers only to the physical map. The consensus map produced by DAGGER is generated by linearizing an unsimplified consensus graph that is equivalent to the linkage maps. By contrast, the consensus map produced by MergeMap is generated by linearizing a simplified consensus graph that is consistent with but not equivalent to the linkage maps. Based on the linkage maps in Table 2, the hardness locus should be less than 1 cM in length; this is true in the DAGGER map but not the consensus map from MergeMap.

**Figure 4:**
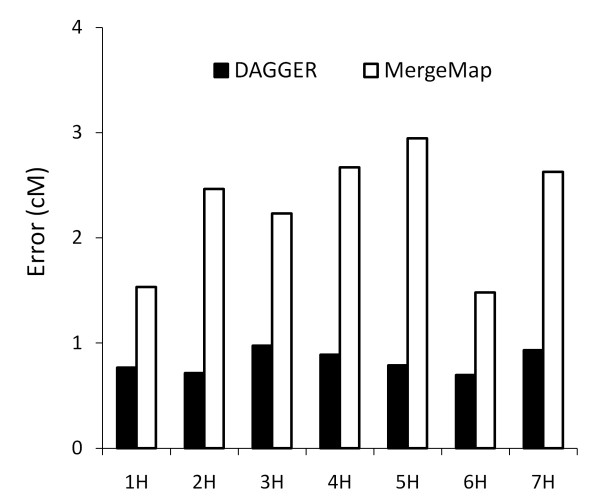
**RMS error between the consensus map and barley linkage maps**. The SNP consensus map from DAGGER had consistently less error than the consensus map from MergeMap. When averaged over the seven chromosomes, the RMS error (square root of normalized Equation 1) was 0.82 cM per marker interval with DAGGER compared to 2.28 cM with MergeMap.

Nonetheless, there were several indications of improved fine structure with DAGGER. Figure 3 compares the two (rescaled) consensus maps at the 5HS telomere. On the physical map the hardness locus (*hinb *to *gsp*) is 110 kb, within which there was only 1 recombinant out of the 278 total individuals in the SM, OWB, and MB populations [11]. This is reflected in the linkage maps in Table 2, where all the hardness locus markers appear in one bin in both the SM and MB linkage maps and in two bins separated by 1.1 cM in the OWB linkage map. From these data the hardness locus should occupy less than 1 cM in the consensus map, which was true with DAGGER (0.8 cM interval) but not with MergeMap (4.2 cM interval). Figure 4 shows the RMS error between the consensus map and the linkage maps for each of the seven barley chromosomes. The DAGGER map had consistently less error, with an average of 0.82 cM per marker interval compared to 2.28 cM with MergeMap.

## Conclusions

New algorithms for the simplification and linearization of consensus graphs have been implemented as a package for the R computing environment called DAGGER. The package offers both linear and quadratic programming options for linearizing the consensus graph. When these two methods were compared using simulated data, the consensus maps generated by quadratic programming had less compression and higher accuracy. When applied to four barley linkage maps genotyped at nearly 3000 SNP markers, in less than one minute DAGGER produced a consensus map with improved fine structure compared to the existing barley consensus SNP map. The RMS error between the linkage maps and the DAGGER map was 0.82 cM per marker interval compared to 2.28 cM for the existing consensus map. Examination of the barley hardness locus at the 5HS telomere confirmed that the DAGGER output was more accurate for fine structure analysis. DAGGER is an effective, freely available resource for integrating the information from a set of consistent linkage maps.

## Methods

### Linearizing the consensus graph

DAGGER uses the R packages quadprog [27] and Rglpk [28] for the QP (Equation 4) and LP (Equation 5) options, respectively. The package quadprog is only for strictly convex quadratic programs, i.e., those in which the quadratic form is positive definite. Because the adjacency matrix **A **is for an acyclic graph, the addition of row vector [1 0 0 ... 0] makes a full column rank matrix **Ã** (the corresponding entry in **d **is an arbitrary constant, say zero, which fixes the origin of the consensus map). With this addition, the combined adjacency matrix  (formed by stacking **Ã **on **B**) is also full column rank, and the quadratic form is positive definite 

### Validation with simulated data

To simulate *g *linkage maps, 2*g *parents with a fixed average level of sequence identity were randomly generated and paired to create *g *doubled haploid populations. For each population, gamete formation was simulated for 200 individuals assuming no crossover interference. Linkage maps were constructed using Haldane's mapping function and a LOD-score weighted-least-squares approach [2,29]. The additional constraint of fixed marker order (equal to the simulated physical order) was used to create linkage maps with no ordering conflicts. The results in this manuscript are based on four-locus linkage maps; changing to three or five loci had little effect.

Statistical analysis was conducted using PROC MIXED in SAS 9.2 (SAS Institute, Cary, NC), with each simulation as "subject" to properly model the covariance structure (LP and QP consensus maps were generated for each simulation).

### Validation with barley data

When the four linkage maps published by Close et al. [11] were used as input for DAGGER, no ordering conflicts were detected. Markers from unassigned linkage groups were not included, which led to three fewer markers (3_0024, 3_0764, 2_1056) in the consensus map of chromosome 4H compared with the results of Close et al. [11]. The DAGGER consensus map for each chromosome took several seconds to generate on a laptop computer running R 2.12.1 [22]. Following the procedure of Close et al. [11], the consensus maps were rescaled (by chromosome) to equal the mean length of the four linkage maps.

Comparisons were made to both the published results of Close et al. [11] as well as *de novo *results generated by submitting the four linkage maps to MergeMap Online [16] on 23 Feb. 2011 (followed by rescaling). The comparisons were nearly identical, e.g., the RMS error for the map of Close et al. [11] was 2.23 cM vs. 2.28 cM for the *de novo *MergeMap output. The results in this manuscript are for the *de novo *MergeMap output.

The physical map in Figure 3 is based on GenBank accession number AH014393.1[21]. The positions of the SNP markers on the physical map were determined using BLAST 2 Sequences [30], with the query sequences taken from Close et al. [11]. The consensus map illustrations in Figure 3 were created using MapChart [31].

## Abbreviations

DAG: directed acyclic graph; LP: linear program(ming); QP: quadratic program(ming); RMS: root-mean-squared; SNP: single nucleotide polymorphism; SE: standard error.

## Supplementary Material

Additional file 1 **Figure S1**. Consensus graph for chromosome 1H. Click here for file

Additional file 2 **Figure S2**. Consensus graph for chromosome 2H. Click here for file

Additional file 3 **Figure S3**. Consensus graph for chromosome 3H. Click here for file

Additional file 4 **Figure S4**. Consensus graph for chromosome 4H. Click here for file

Additional file 5 **Figure S5**. Consensus graph for chromosome 5H. Click here for file

Additional file 6 **Figure S6**. Consensus graph for chromosome 6H. Click here for file

Additional file 7 **Figure S7**. Consensus graph for chromosome 7H. Click here for file

Additional file 8 **Table S1**. Consensus map for the entire genome. Click here for file

## References

[B1] Beavis WD Grant D A linkage map based on information from four F2 populations of maize (*Zea mays *L.) Theor Appl Genet 1991 82 636 644 10.1007/BF00226803 24213346

[B2] Stam P Construction of integrated genetic linkage maps by means of a new computer package: JoinMap Plant J 1993 3 739 744 10.1111/j.1365-313X.1993.00739.x

[B3] Somers DJ Isaac P Edwards K A high-density microsatellite consensus map for bread wheat (*Triticum aestivum L*.) Theor Appl Genet 2004 109 1105 1114 10.1007/s00122-004-1740-7 15490101

[B4] Mace ES Rami J-F Bouchet S Klein PE Klein RR Kilian A Wenzl P Xia L Halloran K Jordan DR A consensus genetic map of sorghum that integrates multiple component maps and high-throughput Diversity Array Technology (DArT) markers BMC Plant Biol 2009 9 13 10.1186/1471-2229-9-13 19171067 PMC2671505

[B5] Danan S Veyrieras J-B Lefebvre V Construction of a potato consensus map and QTL meta-analysis offer new insights into the genetic architecture of late blight resistance and plant maturity traits BMC Plant Biol 2011 11 16 10.1186/1471-2229-11-16 21247437 PMC3037844

[B6] Rostoks N Mudie S Cardle L Russell J Ramsay L Booth A Svensson JT Wanamaker SI Walia H Rodriguez EM Hedley PE Liu H Morris J Close TJ Marshall DF Waugh R Genome-wide SNP discovery and linkage analysis in barley based on genes responsive to abiotic stress Mol Genet Genomics 2005 274 515 527 10.1007/s00438-005-0046-z 16244872

[B7] Wenzl P Li H Carling J Zhou M Raman H Paul E Hearnden P Maier C Xia L Caig V Cakir M Poulsen D Wang J Raman R Smith KP Muehlbauer GJ Chalmers KJ Kleinhofs A Huttner E Kilian A A high-density consensus map of barley linking DArT markers to SSR, RFLP and STS loci and agricultural traits BMC Genomics 2006 7 206 10.1186/1471-2164-7-206 16904008 PMC1564146

[B8] Marcel TC Varshney RK Barbieri M Jafary H de Kock MJD Graner A Niks RE A high-density consensus map of barley to compare the distribution of QTLs for partial resistance to *Puccinia hordei *and of defence gene homologues Theor Appl Genet 2007 114 487 500 10.1007/s00122-006-0448-2 17115126

[B9] Stein N Prasad M Scholz U Thiel T Zhang H Wolf M Kota R Varshney RK Perovic D Grosse I Graner A A 1,000-loci transcript map of the barley genome: new anchoring points for integrative grass genomics Theor Appl Genet 2007 114 823 839 10.1007/s00122-006-0480-2 17219208

[B10] Varshney RK Marcel TC Ramsay L Russell J Röder MS Stein N Waugh R Langridge P Niks RE Graner A A high density barley microsatellite consensus map with 775 SSR loci Theor Appl Genet 2007 114 1091 1103 10.1007/s00122-007-0503-7 17345060

[B11] Close TJ Bhat PR Lonardi S Wu Y Rostoks N Ramsay L Druka A Stein N Svensson JT Wanamaker S Bozdag S Roose ML Moscou MJ Chao S Varshney RK Szücs P Sato K Hayes PM Matthews DE Kleinhofs A Muehlbauer GJ DeYoung J Marshall DF Madishetty K Fenton RD Condamine P Graner A Waugh R Development and implementation of high-throughput SNP genotyping in barley BMC Genomics 2009 10 582 10.1186/1471-2164-10-582 19961604 PMC2797026

[B12] Alsop BP Farre A Wenzl P Wang JM Zhou MX Romagosa I Kilian A Steffenson BJ Development of wild barley-derived DArT markers and their integration into a barley consensus map Mol Breeding 2011 27 77 92 10.1007/s11032-010-9415-3

[B13] Jansen J de Jong AG van Ooijen JW Constructing dense genetic linkage maps Theor Appl Genet 2001 102 1113 1122 10.1007/s001220000489

[B14] Wu Y Close TJ Lonardi S Accurate construction of consensus genetic maps via integer linear programming IEEE/ACM Transactions on Computational Biology and Bioinformatics 2011 8 381 394 10.1109/TCBB.2011.16 20479505

[B15] Yap IV Schneider D Kleinberg J Matthews D Cartinhour S McCouch SR A graph-theoretic approach to comparing and integrating genetic, physical and sequence-based maps Genetics 2003 165 2235 2247 14704199 10.1093/genetics/165.4.2235 PMC1462874

[B16] MergeMap Online http://mergemap.org

[B17] Hamblin MT Close TJ Bhat PR Chao S Kling JG Abraham KJ Blake T Brooks WS Cooper B Griffey CA Hayes PM Hole DJ Horsley RD Obert DE Smith KP Ullrich SE Muehlbauer GJ Jannink J-L Population structure and linkage disequilibrium in U.S. barley germplasm: Implications for association mapping Crop Sci 2010 50 556 566 10.2135/cropsci2009.04.0198

[B18] Roy JK Smith KP Muehlbauer GJ Chao S Close TJ Steffenson BJ Association mapping of spot blotch resistance in wild barley Mol Breeding 2010 26 243 256 10.1007/s11032-010-9402-8 PMC2908432 20694035

[B19] Cuesta-Marcos A Szücs P Close TJ Filichkin T Muehlbauer GJ Smith KP Hayes PM Genome-wide SNPs and re-sequencing of growth habit and inflorescence genes in barley: Implications for association mapping in germplasm arrays varying in size and structure BMC Genomics 2010 11 707 10.1186/1471-2164-11-707 21159198 PMC3018479

[B20] Barley Coordinated Agricultural Project (CAP) http://barleycap.org

[B21] Caldwell KS Langridge P Powell W Comparative sequence analysis of the region harboring the hardness locus in barley and its colinear region in rice Plant Physiol 2004 136 1 14 10.1104/pp.104.044081 PMC523377 15466237

[B22] R: A language and environment for statistical computing http://www.R-project.org/

[B23] R package DAGGER http://cran.r-project.org/web/packages/DAGGER/

[B24] Cormen TH Leiserson CE Rivest RL Stein C Introduction to Algorithms 2009 3 Cambridge: MIT Press

[B25] Graphviz dot http://graphviz.org

[B26] Boyd S Vandenberghe L Convex Optimization 2004 Cambridge: Cambridge University Press

[B27] R package quadprod http://cran.r-project.org/web/packages/quadprog/

[B28] R package Rglpk http://cran.r-project.org/web/packages/Rglpk/

[B29] Liu BH Statistical Genomics 1998 Boca Raton: CRC Press

[B30] BLAST 2 Sequences http://www.ncbi.nlm.nih.gov/blast/bl2seq/wblast2.cgi

[B31] Voorrips RE MapChart: Software for the graphical presentation of linkage maps and QTLs J Heredity 2002 93 77 78 10.1093/jhered/93.1.77 12011185

