# Supplementary figures and images for: New algorithm improves fine structure of the barley consensus SNP map
Source: BMC Genomics. 2011 Aug 10;12:407. doi: 10.1186/1471-2164-12-407 (PMC3179964; doi:10.1186/1471-2164-12-407)

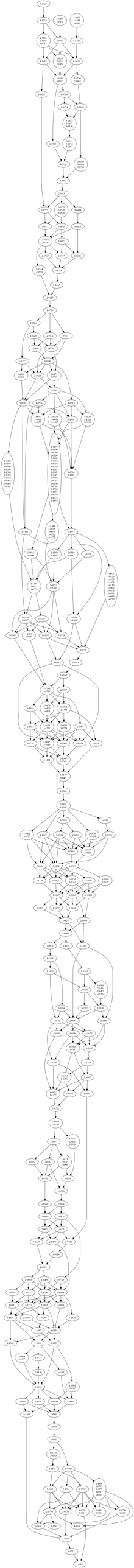

Supplement: Additional file 1 — Figure S1. Consensus graph for chromosome 1H. [file 1471-2164-12-407-S1.JPEG]

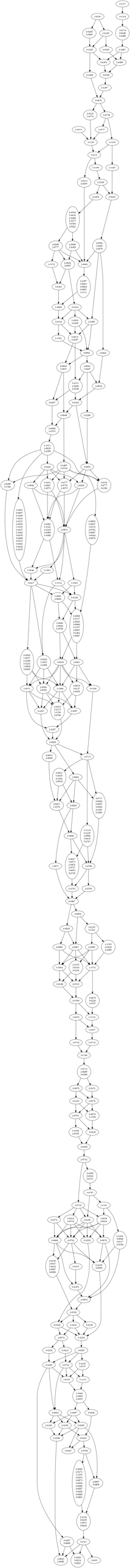

Supplement: Additional file 4 — Figure S4. Consensus graph for chromosome 4H. [file 1471-2164-12-407-S4.JPEG]

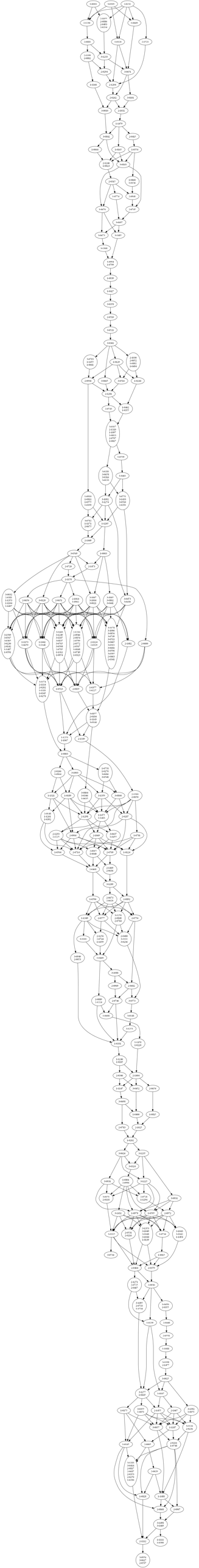

Supplement: Additional file 6 — Figure S6. Consensus graph for chromosome 6H. [file 1471-2164-12-407-S6.JPEG]

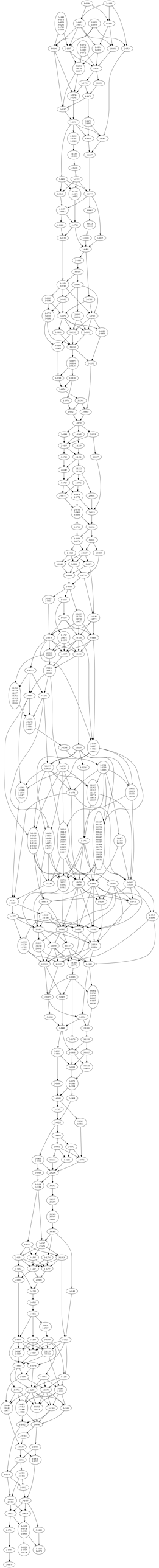

Supplement: Additional file 7 — Figure S7. Consensus graph for chromosome 7H. [file 1471-2164-12-407-S7.JPEG]
